# Generalizability and effect size of the impact of uncontrolled hypertension in patients with type 2 diabetes mellitus

**DOI:** 10.1111/jch.14471

**Published:** 2022-03-21

**Authors:** Kristy Bono, Dhvani Shihora, Anurag Modak

**Affiliations:** ^1^ Department of Medicine New Jersey Medical School Rutgers University Newark New Jersey USA; ^2^ Center for Advanced Biotechnology and Medicine Robert Wood Johnson Medical School Rutgers University Piscataway New Jersey USA

**Keywords:** ADA guidelines, hypertension, type 2 diabetes mellitus

We read with pleasure the article by Rabizadeh et al., titled “*Uncontrolled hypertension in patients with type 2 diabetes: What are the correlates*,” which determined that body‐mass index (BMI), pulse pressure, total cholesterol, and non‐HDL cholesterol are significant predictors of uncontrolled hypertension in patients with type 2 diabetes mellitus (T2DM).^1^ In addition, ineffective monotherapy, medical inertia, and patient non‐compliance were other contributors to the uncontrolled hypertension.^1^ However, the following limitations should be considered when interpreting the results of this study.

First and foremost, this study provided a loose definition of uncontrolled hypertension. Researchers characterized uncontrolled hypertension as patients who were measured as having a systolic blood pressure (SBP) >140 mmHg or diastolic blood pressure (DBP) >90 mmHg in their first visit of the study.^1^ Although the authors referenced the American Diabetes Association's (ADA) 2019 guidelines as basis for this approach, the ADA guidelines state that a diagnosis of hypertension should be given for SBP >140 mmHg and DBP >90 mmHg during separate visits.^2^, ^3^ Therefore, with the researchers’ definition, patients may have been included in the uncontrolled hypertension group who did not meet the criteria under the ADA guidelines. These guidelines are important, because the BP measurement during a single visit is an unreliable indicator of hypertension.^4^ Many factors can cause temporary elevation of BP, such as white coat hypertension, stress, and physical activity.^4^ Therefore, it is possible that the study's uncontrolled hypertension group contained normotensive patients and, as a result, the data underestimated the degree to which certain factors predict uncontrolled hypertension in diabetics.

Additionally, the generalizability of this study to a broader population is limited given that it was performed at a single diabetes clinic in Iran. Although the study population was large, this study failed to demonstrate the impact of the physical, cultural, and socioeconomic factors that has been shown to impact high BP and T2DM.^5^, ^6^ Low socioeconomic status and low educational attainment have been linked to increased levels of high BP.^5^ Further, demographics, access to healthcare, socioeconomic status, and psychological distress have been determined to be major predictors of mortality in those with diabetes.^6^ These external factors will vary across nations and across the world. Therefore, by focusing on the biological predictors of uncontrolled hypertension in diabetics, the authors missed important external factors.

Moreover, the study excluded patients younger than the age of 30 years, which further reduces the generalizability of the results. Commonly, in studies examining T2DM, the inclusion criteria are age >18 years.^7^, ^8^, ^9^ However, the researchers neglected to include the young adult population. This exclusion criteria may have caused certain factors that are more prevalent in the older population to appear as more statistically‐significant predictors than what would otherwise be representative of the entire adult population with T2DM.^10^, ^11^, ^12^ In fact, younger patients are less likely to develop high BP, but have a rising prevalence and incidence of obesity, and subsequently T2DM.^10^, ^11^, ^12^ Thus, the authors are overextending the applicability of their results, which may only hold true for the older population of adults with T2DM in Iran, but are not representative of all adult patients with T2DM.

Despite potential limitations mentioned above, we believe that the authors developed a strong case for the association of various factors, such as BMI, pulse pressure, and triglycerides, as predictors for the development of uncontrolled hypertension in those with T2DM. The study indicates the need for further research in this area.

## CONFLICT OF INTEREST

The authors declare that they have no conflicts of interests.
